# Immunologic Considerations in Heart Transplantation for Congenital Heart Disease

**DOI:** 10.2174/157340311797484204

**Published:** 2011-05

**Authors:** Beth D Kaufman, Robert E Shaddy

**Affiliations:** From the Children’s Hospital of Philadelphia, and the University of Pennsylvania School of Medicine, Philadelphia, PA

**Keywords:** Heart transplantation, allosensitization, rejection, pediatric, therapy, management.

## Abstract

Children and adults with congenital heart disease (CHD) can require interventions that result in immunologic alterations that are different than those seen in patients with cardiomyopathies. Patients with CHD can be exposed to heart surgeries, blood products, valved and non-valved allograft tissue, and mechanical circulatory support, all of which can alter the immunologic status of these patients. This change in immunologic status is most commonly manifested as the development of anti-human leukocyte antigen (HLA) antibodies. This review will delineate a) the causes of anti-HLA anti-body production (often referred to as allosensitization); b) preventive strategies for anti-HLA antibody production before transplantation; c) treatment strategies for those patients who develop anti-HLA antibodies before transplantation; d) consequences of HLA allosensitization after transplantation; and e) treatment of HLA allosensitization and antibody-mediated rejection after transplantation.

## CAUSES OF ANTI-HLA ANTIBODY PRODUCTION

Anti-HLA antibodies usually can be detected in the blood of individuals in response to an exposure to foreign antigens that stimulate their production. Various stimuli have been identified that result in the development of anti-HLA antibodies. These include pregnancy, transfusions of blood products, valved and non-valved allograft material (including whole organ transplant), and use of ventricular assist devices. Blood transfusions cause anti-HLA antibody production through exposure to antigenic stimuli such as white blood cells and/or platelets. Using filters and/or irradiation reduces the risk of HLA allosensitization, but does not appear to be entirely protective. Patients who undergo repair of CHD, especially if requiring cardiopulmonary bypass, usually are exposed to blood products either during or after surgery. Many patients with CHD, particularly those with complex CHD, often require multiple surgeries throughout their lifetimes, and therefore are exposed to multiple blood products and therefore multiple antigenic stimuli. Valved and non-valved allograft material is commonly used for the repair of CHD. Valved allograft material is used for repairs in the right and left ventricular outflow tracts, and much less commonly in the atrioventricular valves. Non-valved allograft material is used primarily for aortic arch and pulmonary artery reconstruction, in addition to right and left ventricular outflow tracts. Regardless of its site of repair, allograft material used at the time of surgery provides a strong stimulus to the development of anti-HLA antibodies. This response occurs within weeks of implantation and can be detected as circulating very broad reactivity of anti-HLA antibodies for many years [1-5].

## PREVENTIVE STRATEGIES FOR ANTI-HLA ANTIBODY PRODUCTION BEFORE TRANSPLANTATION

Two basic strategies have been tried to prevent the development of anti-HLA antibodies in these situations: 1) the reduction and/or alteration of the antigenic stimulus, or 2) the alteration of the immune response of the individual to the antigenic stimulus. As stated above, using filters and/or irradiation for blood products may decrease the antigenic exposure to an individual, and the mechanism is thought to be primarily through the removal of allosensitizing white blood cells in blood products. Attempts to alter the antigenic load that is presented by allograft material has generally been directed toward removal of antigen-presenting cells on allograft material prior to implantation. These decellularized allografts have been shown to markedly reduce the degree of allosensitization seen after allograft implantation in both animals and in humans, but questions still remain as to the durability and safety of these altered allografts [6-9]. 

Methods to alter the immune system before or during allograft implantation has also met with some degree of success. In animal studies, drugs like cyclosporine and mycophenolic mofetil (MMF) have been successfully used to reduce the alloantibody response to allograft implantation [10, 11]. Peri-operative MMF (but not azathioprine) has also been shown to reduce the response to allograft implantation in children undergoing surgery for CHD [12-14]. Presumably, if patients with CHD were heavily immunosuppressed at the time of allograft implantation for repair of CHD (similar to heart transplantation), there would be significant reduction in the incidence and breadth of allosensitization. However, this level of immunosuppression carries with it significant risks including infectious and neoplastic complications, in addition to all of the drug-specific complications such as renal dysfunction, diabetes, etc. One of the factors limiting the application of all forms of immunosuppression after surgical allograft implantation for CHD is the lack of convincing evidence that the anti-HLA antibody response to the allograft alters its function and/or longevity. Thus, it is difficult to justify routine immunosuppression in children with CHD who receive allograft material at the time of surgery.

The consequences of the development of anti-HLA antibodies in patients with CHD are a matter of debate [15]. Although one would theorize that the presence of donor-specific anti-HLA antibodies has potential for immune-mediated damage to the implanted allograft, it has never been conclusively proven that this is true. Some pathologic studies of explanted allografts in adults have failed to demonstrate evidence of immune-mediated damage (e.g., “rejection”) [16]. However, evidence of such immune injury in explanted allografts of children with CHD has been shown [17 18]. There do not appear to be any other significant systemic adverse effects from these alloantibodies, unless these patients come to need heart or other solid organ transplantation. It has been well-recognized for many years that patients with circulating anti-HLA antibodies, especially those who have a subsequent incompatible HLA crossmatch because of these antibodies, are at increased risk for rejection and graft loss after transplantation for [19, 20]. Because of this, many centers continue to maintain a policy of requiring a prospective crossmatch (either actual crossmatch between donor and recipient, or virtual crossmatch based upon known HLA donor type and recipient anti-HLA antibodies. In these situations where a prospective crossmatch is required (either actual or virtual), wait times for organs are significantly increased due to the resultant reduction in potential donors, and therefore waitlist mortality is significantly increased. In pediatrics, wait times to transplant and mortality after listing have been shown to be double those who do not require a crossmatch [21]. Because of this, some centers have adopted a policy of not requiring a prospective crossmatch, and performing heart transplants with an incompatible HLA match. Using plasmapheresis, thymoglobulin, and increased immunosuppression, early results have been encouraging, and multicenter studies are underway [22-24].

## TREATMENT STRATEGIES FOR THOSE PATIENTS WHO DEVELOP ANTI-HLA ANTIBODIES BEFORE TRANSPLANTATION

Many strategies have been tried to reduce this allosensitization before transplantation in order to optimize the opportunity for finding and transplanting an HLA-compatible organ. These strategies have included plasmapheresis, a procedure which involves extracorporeal removal and replacement of the entire plasma volume (containing antibodies as well as other proteins such as coagulation factors). Plasmapheresis requires placement of a large bore dual lumen catheter in a large central vein. This is often challenging in young children due to size of blood vessels, lack or minimal vascular access because of venous occlusions from previous catheters, and/or systemic venous anomalies. Exchange transfusions are often performed in children who are too small for vascular catheter placement and plasmapheresis. Additional strategies include immunosuppressant medications (such as cyclophosphamide), intravenous immune globulin (IVIG), rituximab, or some combination of these [25-27]. Most of these strategies have been able to demonstrate at least a modest, although transient, reduction in anti-HLA antibodies in some patients. Unfortunately, none of these strategies appear to result in a sustained reduction; furthermore, there is controversy as to whether this transient reduction in anti-HLA antibodies provides enough improvement in outcomes after transplantation to warrant the exposure to the medications, hospitalizations, and risks of these treatments. 

## CONSEQUENCES OF HLA ALLOSENSITIZATION AFTER TRANSPLANTATION

The presence of circulating anti-HLA antibodies before transplantation is associated with increased risk of rejection, graft vasculopathy, graft dysfunction, and death after transplantation [28-31]. Injury to the graft may be acute with hemodynamic dysfunction or more chronic manifesting as chronic rejection or graft vasculopathy. Donor specific anti –HLA antibodies (DSA) may be preformed due to allosensitization prior to transplant, or develop de novo at any time following transplant. The de novo development of anti-HLA antibodies after heart transplantation correlates with decreased long term survival. Patients with de novo antibodies appearing more than one year following transplantation had the poorest survival; 47% at 15 years compared to 70-71% 15 year survival for those with none or only pre transplant anti-HLA antibodies [32].

Most investigations refer to results of generalized anti-HLA panel reactive antibody (PRA) assays rather than donor specific antibodies for determination of allosensitization status. Data from the United Network for Organ Sharing /Organ Procurement and Transplantation Network (UNOS/ OPTN) shows that elevated pretransplant PRA in adult heart transplant recipients is a strong predictor of rejection within 1 year after transplant and mortality [33]. The potential consequence of DSAs on transplant outcomes in children is less well established, and may not be the same as observed in adults due to putative age-related differences in the maturity of the immune system. The incidence of allosensitized pediatric heart transplant candidates appears to be increasing as our CHD population enjoys improved operative survival, only to eventually develop cardiac dysfunction following palliative repair. Coupled with a persistently limited pediatric donor supply, the impact of DSA on pediatric heart transplant outcomes is a current area of significant research efforts. 

Data available for pediatric heart transplant recipients is generally limited by small sample sizes from single center reports with heterogeneous results due to variations in study design or antibody detection methods. There is a consensus that DSA can be detrimental to graft function, and associated with rejection, though findings related to impact of DSA on graft and patient survival is less uniform [21,24, 34-36]. 

**Fig. (1):**
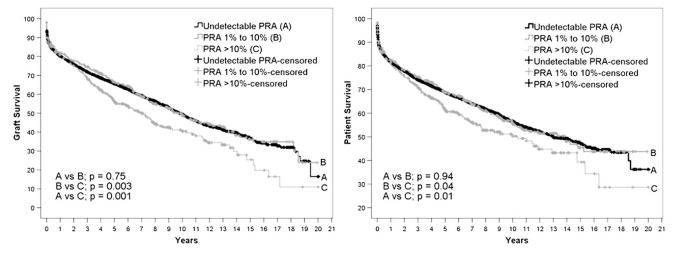
Kaplan–Meier survival curve with log–rank statistics for graft and patient survival. PRA, Panel-reactive antibody. (With permission, 37).

In a recent study from the UNOS registry, pediatric heart transplant recipients with PRA >10% had significantly worse graft and patient survival than those with none or PRA of 1%-10% [37]. Findings were independent of pretransplant status, year of transplantation, donor factors, crossmatch status, and rejection episodes in the first year after transplant. Fig. **1** [37].

There is increasing evidence that anti-HLA antibodies (elevated PRA) are involved in cardiac allograft injury, both acute and chronic, referred to as humoral or antibody-mediated rejection (AMR). Diagnostic criteria for AMR are controversial; however they currently include clinical evidence of cardiac graft dysfunction, donor- specific anti HLA antibodies in the serum, and pathologic findings on endomyocardial biopsy. These pathologic findings may include one or both of the following: histologic abnormalities (capillary injury with endothelial cell swelling, intravascular macrophage and/or neutrophil accumulation) as well as immunostaining abnormalities (immunofluorescent deposits of immunoglobulin and complement diffusely in the vasculature) [38, 39]. 

The most severe form of AMR is referred to as hyperacute rejection which occurs in the first hours after transplant, precipitated by the presence of pre-formed DSA in the circulation that activates the complement system resulting in severe lytic injury to the endothelium and the graft, resulting in severe dysfunction (similar mechanism as injury due to ABO incompatibility). Chronic complement activation with sublytic injuries may occur in transplant recipients with DSA and/or elevated PRA as an ongoing or intermittent process that results in AMR and graft dysfunction. Acute hemodynamic compromise with increased risk for death, and development of early transplant graft vasculopathy has been observed in both adults and children with AMR that occurs after the immediate postoperative period [31]. The development of AMR has a significant negative impact on 15 year graft survival, with only 16% survival in AMR(+) patients compared to 63% survival in those without AMR in one study [40]. Of the 23 patients with AMR in this study, 21 displayed cytotoxic DSA at the time of diagnosis. While the presence of DSA and elevated PRA have been associated with detrimental outcomes, the mechanism has not been directly studied in most clinical investigations. There is some debate as to the uniform ability of all anti HLA antibodies to activate the complement system and cause graft injury. The potential for accommodation has also been considered.

## TREATMENT OF HLA ALLOSENSITIZATION AND ANTIBODY-MEDIATED REJECTION AFTER TRANSPLANTATION

Treatment of antibody – mediated rejection following heart transplant is focused on elimination of circulating antibodies, inhibition of circulating antibodies, suppression of B cells, plasma cell depletion, and/or complement inhibition, in addition to support of graft function which is often impaired due to immune-mediated injury [41]. Attempts to remove antibody are most commonly performed with plasmapheresis. Sedation may be required for catheter placement, which has risks if the patient is experiencing hemodynamic compromise from AMR. Fluid shifts, calcium and other electrolyte flux, and systemic reactions to blood products used for plasma replacement, are other risks related to plasmapheresis and/or exchange transfusion. Immune apheresis (immunoadsorption) is an emerging modality to specifically remove circulating antibodies and immune complexes. Plasmapheresis is often accompanied by the use of high-dose IVIG for immunomodulation to block anti-HLA antibody activity and inhibit complement, as well as corticosteroids to further attenuate the negative effect of circulating antibodies. Therapies to specifically target B cells are often incorporated into treatment for AMR. Rituximab is a chimeric murine/human anti CD20 monoclonal antibody that is utilized to deplete B cells and interfere with antigen-presenting cell (APC) activity to attempt to reduce the risk of recurrent AMR. While most reports have shown utility in treating AMR, all are small case series with different response rates and treatment protocols, combining various doses of rituxumab with IVIG, steroid and plasmapheresis. Serious infections are a notable side effect observed with rituximab therapy [42]. New therapies are constantly being sought since the current methods are not universally successful. Bortezomib is an example of a proteosome l inhibitor that has anti plasma cell properties and therefore is a promising therapy for AMR. Effectiveness of bortezomib against AMR in kidney transplant recipients has been reported, as well as utility in reducing donor-specific anti-human leukocyte antigen antibody [43]. This therapy remains in early stages of investigation.

Support of the patient with significant cardiac graft dysfunction due to AMR may require intravenous inotropes, (milrinone, dobutamine, dopamine) and even temporary mechanical circulatory support due to acute hemodynamic decompensation. Following an episode of AMR, a change and/ or increase in doses of maintenance immunosuppression is often considered. This may entail replacement of cyclosporine with tacrolimus in patients receiving cyclosporine-based immunosuppression, change in antiproliferative agents from azathioprine to MMF, addition of rapamycin (mammalian target of rapamycin inhibitor), and/ or corticosteroids. 

Cardiac catheterization for assessment of hemodynamics and endomyocardial biopsies including immunohistochemistry examination should be considered within weeks after initiation of AMR therapy to help assess effectiveness of therapy. Graft dysfunction may be prolonged. 

Emergent listing for retransplantation can be considered if the above measures do not restore acceptable cardiac function. However, retransplantation in first 6 months following heart transplant and or for acute rejection is not recommended by the International Society of Heart and Lung Transplant (ISHLT) post transplant guidelines due to poor survival [44].
